# New Literature

**DOI:** 10.1155/S1463924680000144

**Published:** 1980


the use of Directional Dust Gauges to monitor air pollution in and around a steel works. The gauge, which is cheap and uses no power, was described and the effects of wind direction and speed, gauge orientation and height above ground level on the data obtained were discussed. Mr Snowsill [5] described two devices which had been developed to monitor emissions from power stations; one used the attenuation of a light beam to give a measure of the dust in a stack emission, the other worked on an optical back-scattering system. The advantages and disadvantages of these devices were discussed and much useful information given on ways of eliminating contamination and dirtying of the optical system. Finally,, Mr Lillenfeld [6] described a number of monitoring instruments developed by his company. He paid particular attention to a silicon photo-detector optical instrument which automatically monitored light scattered from dust (viz. SIMSLIN, described by the first speakers) and a fibrous aerosol monitor for asbestos monitoring which used a combination of electrical alignment of the fibres, followed by optical detection. The calibration and performance of this monitor was compared to the standard NIOSH optical-counting filter method.

The meeting was very interesting, with a wide cross-section of ideas being presented, and was much appreciated by an audience of just under 100; an attendance which was gratifying to the organisers and speakers alike.

REFERENCES

[1] 'The Design and Field Use of SIMSLIN-II - a Continuously Recording Dust Sampling Instrument' by Dr M J Leck and Dr D B Blackford (Research and Laboratory Services Division, Health and Safety Executive, Sheffield).

[2] 'Source Monitoring Systems for Particulates' by Mr S C Wallin (Warren Spring Laboratory, Stevenage).

[3] 'Monitor for Low Level Dust Emissions' by Mr R I Ross (Unilever Ltd., Engineering Division, London).

[4] 'The Application of Directional Dust Gauges to Airborne Dust Monitoring at British Steel's Redcar Development' by Dr B E Prater (British Steel Corporation, Teeside Laboratories).

[5] CEGB, Experience of On-stream Dust Monitoring' by Mr W L Snowsill (Central Electricity Research Laboratories, Leatherhead).

[6] 'Asbestos Monitoring Instrumentation' by Mr P Lillenfeld (G.C.A. Technology, Massachusetts, USA).

*Clive J. Jackson*

### New Literature

### Liquid chromatography application

'The determination of herbicide photolysis products by LC/MS' by R. F. Skinner, Q. Thomas, J. Giles and D. G. Crosby, is a recent publication from the Finnigan Corporation. Oryzalin, a pre-emergency herbicide, and its degradation products have been analyzed by Liquid chromatography/mass spectrometry (LC/MS). This study illustrates the use of Finnigan's belt type interface in LC/MS analysis.

*Copies of the report are available from Finnigan Corporation, 845 West Maude Ave, Sunnyvale, California 94086, USA.*

### Densitometer

The SD 3000 spectrodensitometer from Schoeffel Instruments for the quantitative analysis of thin layer chromatograms is described in an 8-page brochure published by the Schoeffel Instrument Division of Kratos. The SD 3000 is a thin layer chromatogram scanner capable of making absorbance and fluorescence measurements of spotted thin layer separations on TLC plates, paper or film as well as agarose gels, disc gels, slab gels and electrophoretic media. The densitometer is modular in design. Accessories include the necessary carriers for various media, and an electronic reporting integrator for thorough quantitative analysis reports.

*The brochure is available from Kratos Inc, Schoeffel Instrument Division, 24 Booker St, Westwood, NJ 07675, USA.*

### Spectroscopic study of liquids and gases

This report, 'A CARS system for the study of liquids and gases' by D. R. Williams and I A. Stenhouse details the technique of coherent anti-strokes raman spectroscopy (CARS). The basic theory of the technique is given and an experimental CARS system for the study of liquids and gases is described. The system was commissioned by observing the CARS spectra of liquids.

*Copies of the report, price £1.00, are available from HMSO, 49 High Holborn, London WC1V 6HB, UK.*

### Liquid chromatography

High performance liquid chromatography and its application in pharmaceutical analytical problems is the topic of a new book now available from Hewlett Packard. The 157-page book reviews the use of HPLC for pharmaceutical measurements in terms of applications and methodology. Prime applications such as isolation of natural compounds, pharmacokinetics and drug metabolism are described with sample chromatograms and illustrative figures. Sections on methodology explain sample preparation procedures, separation procedures, separation techniques, detectors and quantitative analysis.

*'High performance liquid chromatography in pharmacuetical analyses' is available free of charge from Hewlett-Packard Ltd, King Street Lane, Winnersh, Wokingham, Berks, UK.*

### Automatic data acquisition

'Automatic data acquisition and processing with the APEX goniometer, PDP 11/03 and IBM 370 computer, with application to surface texture studies of magnox fuel cladding', by M.O. Boles, B.A. Bellamy and G.A. Burras, is the title of a recent publication from HMSO. It takes the form of a working manual enabling the user to operate the described system and make modifications to suit individual requirements. Part one describes the general procedures required for automatic data acquisition and processing. Part two describes in detail the application of automatic data collection to texture studies of magnox fuel cladding.

*Copies of the report are available from HMSO, PO Box 569, London SE1 9NH, price £2.00.*

### Phototitration book

A 160 page book on methods of analysis using photometric end-point sensing has been published by Mettler which covers the theory of titration. Detailed methods are suggested for many metals, including calcium, magnesium, aluminium, etc and for dissolved oxygen, sulphate, fluorine, halogens and vitamin C. The book contains an extensive section giving literature references for analytical methods using phototitration.

*'Phototitration - a guide to methods' by K Mooibroek is available from MSE Scientific Instruments, Manor Royal, Crawley, West Sussex, UK, price £25.00.*

### Gamma-ray spectrum of natural Ti

The capture gamma-ray spectrum of natural Ti 'A note on the capture gamma-ray spectrum of natural Ti produced by thermal neutrons' is the subject of a paper by M G Sowerby of AERE Harwell. The data on the spectrum are reviewed.
*HMSO, 49 High Holborn, London WC1V 6HB, UK.*

### Sulphur analyser

A bulletin, published by Fisher Scientific details how their sulphur analyzer, by employing a specific titration reaction with microprocessor control, provides an instrumental approach to sulphur analysis. It is applicable to all metallurgical products, has a short analysis time and is essentially independent of sample characteristics. Data is provided demonstrating the linearity, accuracy and precision of results obtainable with steel samples.
*Fisher Scientific Co, 711 Forbes Ave, Pittsburgh, PA 15219, USA.*

### Nucleonics accessories brochure

Packard Instrument have published a new brochure giving details on their range of scintillation cocktails, reagents, vials and reference materials for liquid scintillations and automatic gamma counting techniques. The brochure which contains details on over 250 separate items of scintillation counting consumables should be of interest to anyone who uses radioassay techniques in the laboratory.
*Copies of the brochure are available from Packard Instrument Ltd, 13-17 Church Road, Caversham, Berks, UK.*

### Preventative maintenance schemes

Technicon's planned preventative maintenance schemes have been drawn up to provide the users of their equipment with systems which run consistently at maximum performance. Three schemes are available including an annual overhaul, check visits and emergency visits or a loan module service depending which scheme is opted for.
*A leaflet giving further details of the service schemes is available from Compagnie Technicon SA, 39, Boulevard de la Muxette, F-95140 Garages-les-Gonesse, France.*

### Clinical laboratory standards

The 1978 annual report of the National Committee for Clinical Laboratory Standards is now available. The report lists details of the committee, a financial statement and a summary of the 1978 activities. A list of the NCCLS active membership as at 31 January 1979 is given.
*NCCLS, 771 East Lancaster Ave, Villanova, PA 19805, USA.*

### Switches booklet

A leaflet from Techmation describes the Giannini range of Enviro-Switches. The contacts of the switches are enclosed within a welded chamber and are operated externally by a magnetic action. The leaflet describes the available switch materials and such configuration as push-button switches, rotary switches, plungers, key-lock switches and limit switches.
*Copies of the leaflet 'Do your switches switch off when the going gets tough?' are available from Techmation Ltd, 58 Edgware Way, Edgware, Middx, UK.*

### Automation of chemical oxygen demand analyses

A paper is available from Techmation which describes the automatic measurement of chemical oxygen demand by Johnson COD meter in the sewage systems of a Swedish pulp mill. Advantages of the COD meter, including the automation of laboratory work and high measuring capacity are discussed together with the results of correlation studies between BOD and COD analyses for various wastewater types.
*Techmation Ltd, 58 Edgware Way, Edgware, Middx, UK.*

### Printed circuit board components

A free catalogue has been published by Sealectro covering their ranges of pins, jacks and links for printed circuit boards, multi-layer and flex circuits. Intended as a guide to the readily available styles from their existing pin jacks and terminal pin ranges, this catalogue also includes a new range of barb and swage mounting pins.
*Sealectro Ltd, Walton Road, Farlington, Portsmouth Hants, UK.*

### Chromatography newsletter

The August 1979 issue in the continuing series of Chromatography Newsletters is available from Perkin-Elmer. Articles on advanced technology applications in both liquid and gas chromatography are featured in the Newsletter, seven of which in this issue are written by outside laboratories.
*Copies of the newsletter can be obtained from Perkin-Elmer Ltd, Post Office Lane, Beaconsfield, Bucks, UK.*

### News

### National Committee for Clinical Laboratory Standards (NCCLS)

The National Committee for Clinical Laboratory Standards is an American (United States of) organisation that develops and publishes standards for clinical laboratories. By the voluntary implementation of these standards in United States laboratories, it is hoped that the imposition of regulations by outside agencies will be avoided.

The active membership of the committee comes from organisations covering the widest range of interests. The organisations include Industry, Government, Trade Associations, as well as Professional and Learned Societies. The corresponding membership includes hospitals, clinics and medical schools throughout the United States. It is by such wide consultation that consensus standards can be achieved.

The documents published cover a comprehensive range of laboratory problems. These include consideration of hardware requirements, instrument servicing, materials, as well as standards for the evaluation of experiments, calculation of results and the expression of data.

The standards are of three types, Approved, Tentative, and Proposed. Comments are invited from any interested reader and not only from those active in the NCCLS. It is evident that efforts are made to keep the standards as up to date and as relevant as can reasonably be achieved. As well as the revision and republication of existing standards, approximately twenty new standards are to be published in the near future.

All the standards are produced to the same high quality. The documents are set out clearly and the subsections are clearly referenced. Also where relevant, ample bibliography is given. Although intended for the clinical chemist the standards can yield useful data to those with an interest in related fields.

The following documents are pertinent to Automatic Analysis:

